# Supplementary material for: RobusTAD: reference panel based annotation of nested topologically associating domains
Source: Genome Biol. 2025 May 19;26:129. doi: 10.1186/s13059-025-03568-9 (PMC12087246; doi:10.1186/s13059-025-03568-9)
Supplement: Supplementary file 1 — Additional file 1: PDF document containing supplementary notes, figures, and table. [file 13059_2025_3568_MOESM1_ESM.pdf]

Additional file 1
Supplementary Information

Yanlin Zhang[1], Rola Dali[1], and Mathieu Blanchette[1*]

[1]*School of Computer Science, McGill University, Montréal, Québec, H3A 0E9, Canada*
[*]*Correspondence: blanchem@cs.mcgill.ca*

Note S1. A comparison of singleton TADs, TADs, and subTADs

RobusTAD detects TAD hierarchies from Hi-C contact maps, allowing us to classify TAD predictions into distinct categories. These categories include singleton TADs, which are isolated TADs that do not overlap with others; TADs, which are non-singleton TADs that do not reside within larger TADs; and sub-TADs, which are non-singleton TADs found within larger TADs. Sub-TADs are further divided into three groups: sub-TAD A, with left boundaries being left TAD boundaries; sub-TAD B, with right boundaries being right TAD boundaries; and sub-TAD C, encompassing other subTADs. Rescaled pileup plots in Fig. S10a show that within-domain interactions in predicted TADs of all groups are larger than their surroundings. Notably, we observe dot-corners displaying increased interactions across all groups, with the strongest dot-corner pattern associated with TADs. Singleton TADs exhibit relatively weak domain boundaries and less involvement in transcription. These boundaries also show lower enrichment of CTCF binding sites and RAD21. In contrast, TAD boundaries (including right boundaries of subTAD A and left boundaries of subTAD B) serve as robust insulating regions compared to other types of domain boundaries. They are more enriched for architectural proteins such as CTCF and RAD21 and more actively involved in transcriptional processes (as indicated by TSS and ATAC-seq signals around these boundaries). Sub-TAD boundaries (including both boundaries of sub-TAD C, right boundaries of sub-TAD A, and left boundaries of sub-TAD B) are relatively weaker and less enriched for architectural proteins. The presence of Tss at sub-TAD boundaries falls between that of TAD boundaries and singleton TAD boundaries. Additionally, both boundaries of sub-TAD C display greater accessibility than domain boundaries of any other type, as measured by ATAC-Seq. Furthermore, We studied Enhancer-Promoter links by comparing TAD boundary pairs against polII ChIA-PET data (Fig. S10b). We found that 5% of TADs are E-P links; 15%-20% of singleton TADs, sub-TADs A and B are E-P links; and more than 28% of sub-TADs C are E-P links. Fig. S10c illustrates that TAD boundary pairs are notably enriched in convergent CTCF motifs compared to other boundary pairs, with singleton TADs showing less enrichment in convergent CTCF motifs. In summary, these

observations show the importance of TAD hierarchies in facilitating gene expression and regulation, with TADs frequently associated with boundaries marked by convergent CTCF motifs, and subTADs playing an important role in gene regulation, with a substantial portion being E-P links.

Note S2. Refined boundary score is a stratified rank-sum test

In RobusTAD, to refine score of a putative domain boundary b_i, we select samples in the reference panel that have domain boundaries within a 50 kb region around b_i. TAD are relatively conserved across cells. Thus, we assume domain boundaries inside this 50 kb region are identical among all samples (i.e., study sample and selected reference samples), and compute refined boundary scores as the mean boundary scores for the study sample and all selected reference samples. Here, we show that a mean boundary score is equivalent to a stratified rank-sum test score where each stratum contains all interaction frequencies of a particular genome distance that comes from a particular sample. This stratified rank-sum test evaluates the strength of a putative boundary using interaction frequencies from all samples that exhibit similar local structures in our study. Each sample is an observation of the same local structure. To simplify, we consider refined boundary scores as the mean of two samples' scores S^1 and S^2 computed with a window of size w. Boundary scores evaluated from the kth diagonal are $S^{1,k}$ and $S^{2,k}$ respectively. Following our definition of the boundary score, we have

$$S^1 = \frac{1}{w \times w} \sum_{k=1}^{w} w S^{1,k}$$
$$S^2 = \frac{1}{w \times w} \sum_{k=1}^{w} w S^{2,k}$$

thus,

$$\begin{aligned}
\text{mean}(S^1, S^2) &= \frac{1}{2}(S^1 + S^2) \\
&= \frac{1}{2}(\frac{1}{w \times w} \sum_{k=1}^{w} w S^{1,k} + \frac{1}{w \times w} \sum_{k=1}^{w} w S^{2,k}) \\
&= \frac{1}{2 \times w \times w} \sum_{i=1}^{2} \sum_{k=1}^{w} w S^{i,k}
\end{aligned}$$

is a stratified rank-sum test, where $2 \times w \times w$ is the total number of interaction frequencies and w is the number of interaction frequencies in each stratum. (Note that, for improved clarity in explanation, we chose not to cancel out w).

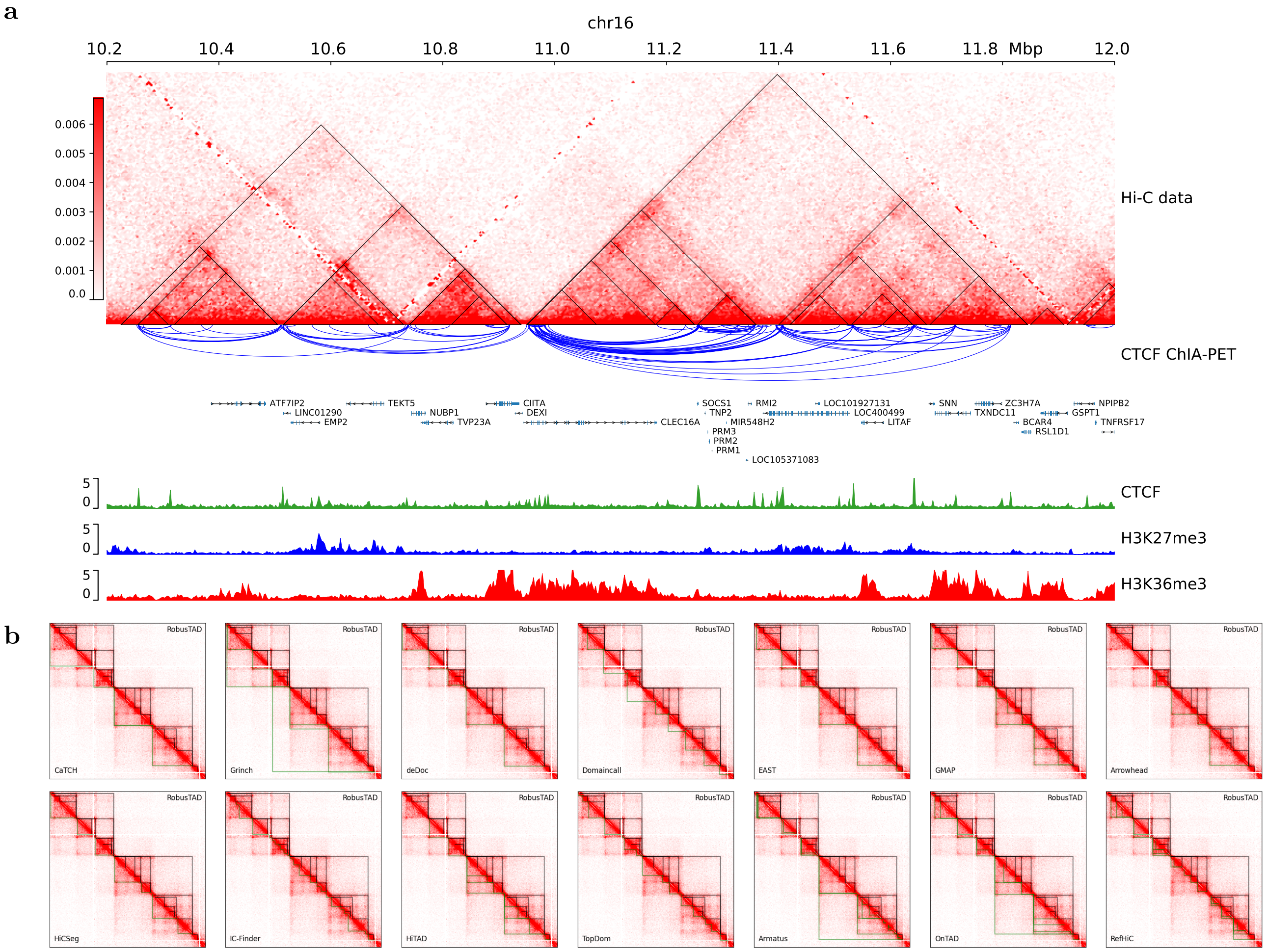

Figure S1. TAD identification for an example genomic region (chr16:10.2 Mb – 12 Mb) of GM12878 cells. a, TAD identified by RobusTAD on GM12878 cells. Note how TAD predictions are supported by the CTCF ChIA-PET data and consistent with gene annotation and epigenetic features. **b,** Comparison of TADs detected by different tools. RobusTAD annotated two nested sets of TADs. Every gene in this region is included entirely within a TAD. Most predicted TAD boundaries are collocated with ChIP-seq peaks, and loops identified by CTCF ChIA-PET support many TAD predictions; both support the conclusion that RobusTAD produces accurate TAD annotations. We also observed that TADs annotated by RobusTAD are either enriched for either activation (H3K36me3) or repression (H3K27me3) marks, but rarely both. ChIA-PET data suggests that a weak TAD (chr16:10.25Mb-10.7Mb) is missed by RobusTAD because it partially overlaps other TADs. Among all tools, only CaTCH detected this weak TAD.

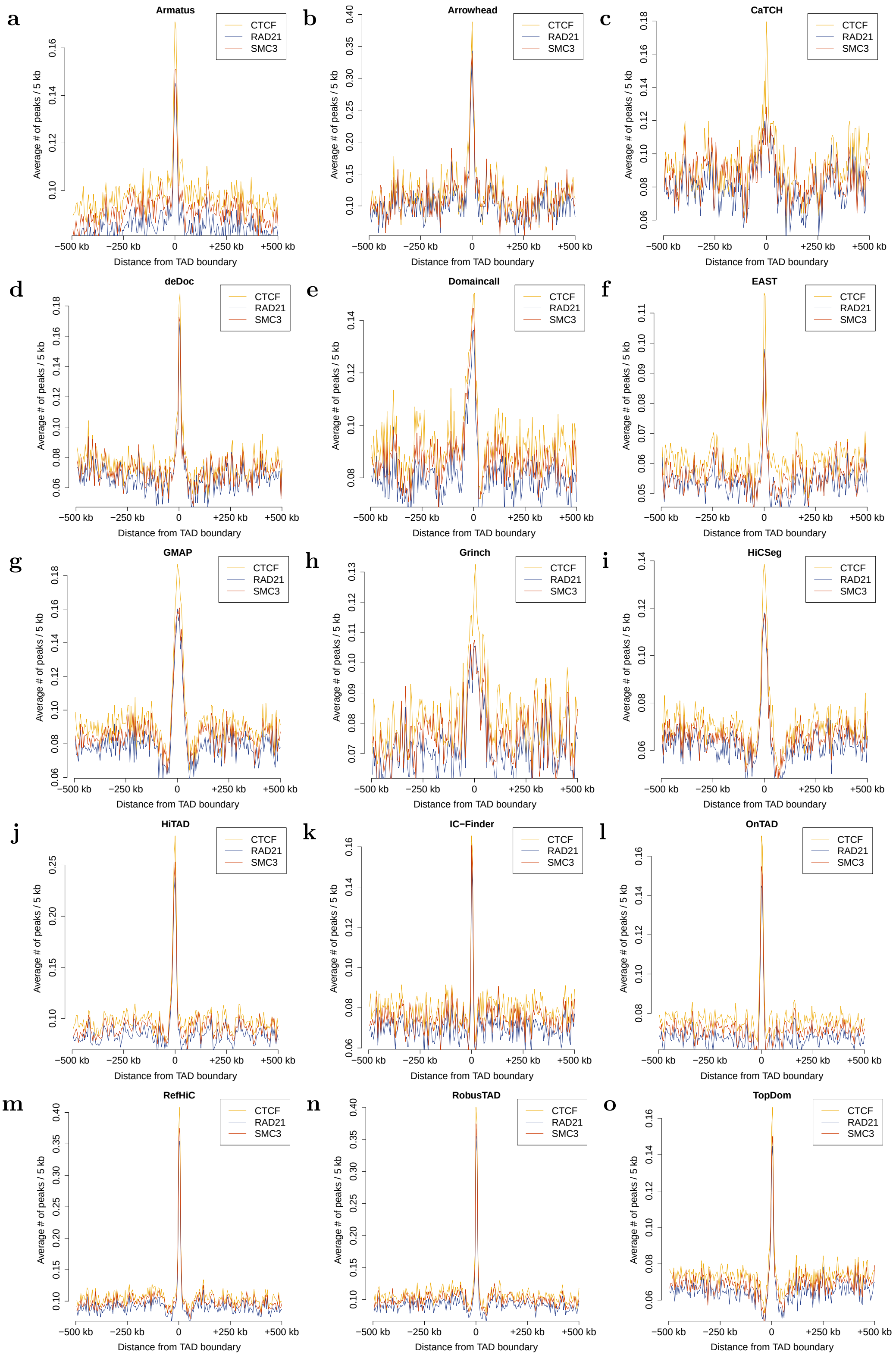

Figure S2. ChIP-seq peak signals for CTCF, RAD21, and SMC3 around TAD boundaries annotated by each tool.

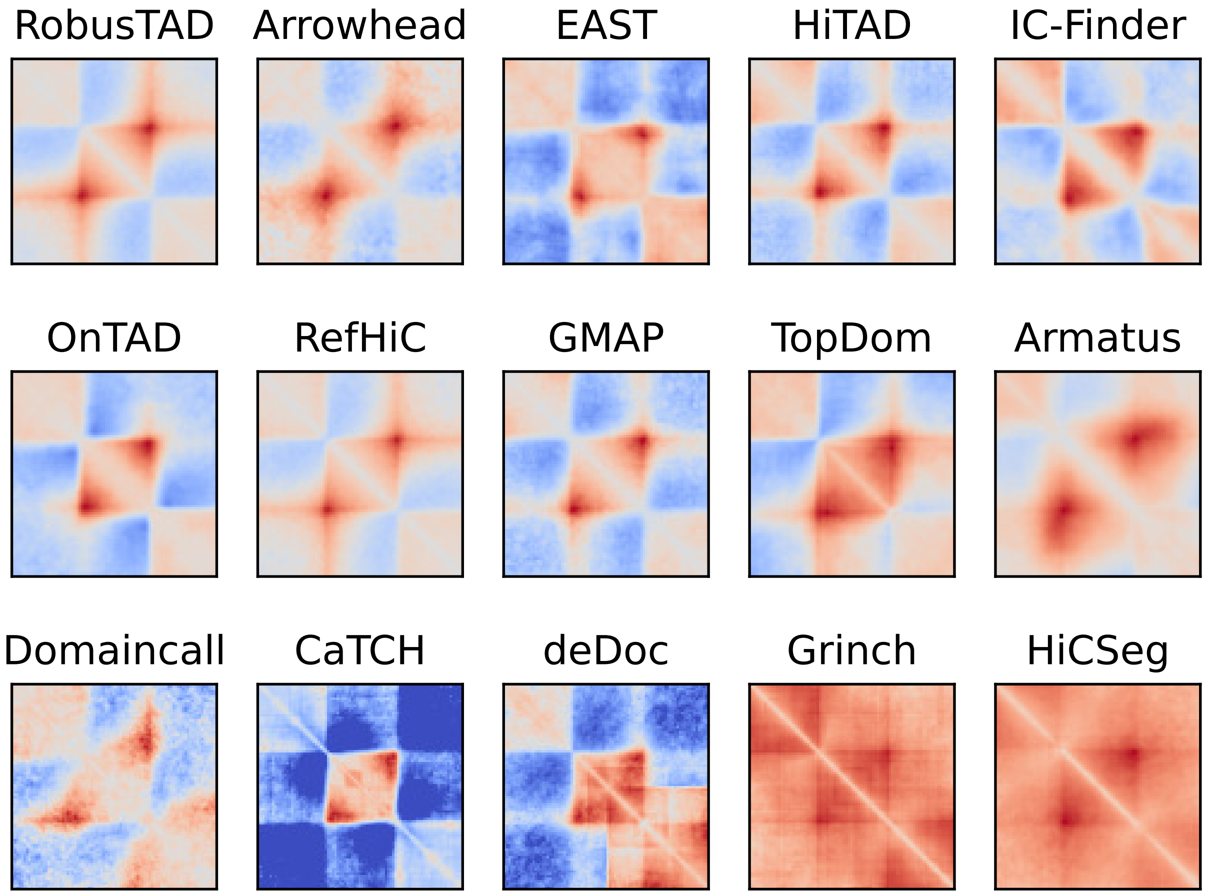

Figure S3. Visual comparison of TADs predicted by RobusTAD and 14 other tools from a GM12878 Hi-C data. These plots are created by aggregating regions over a Hi-C contact map containing 250M valid read pairs. The regions are TAD predictions used in Figure 3.

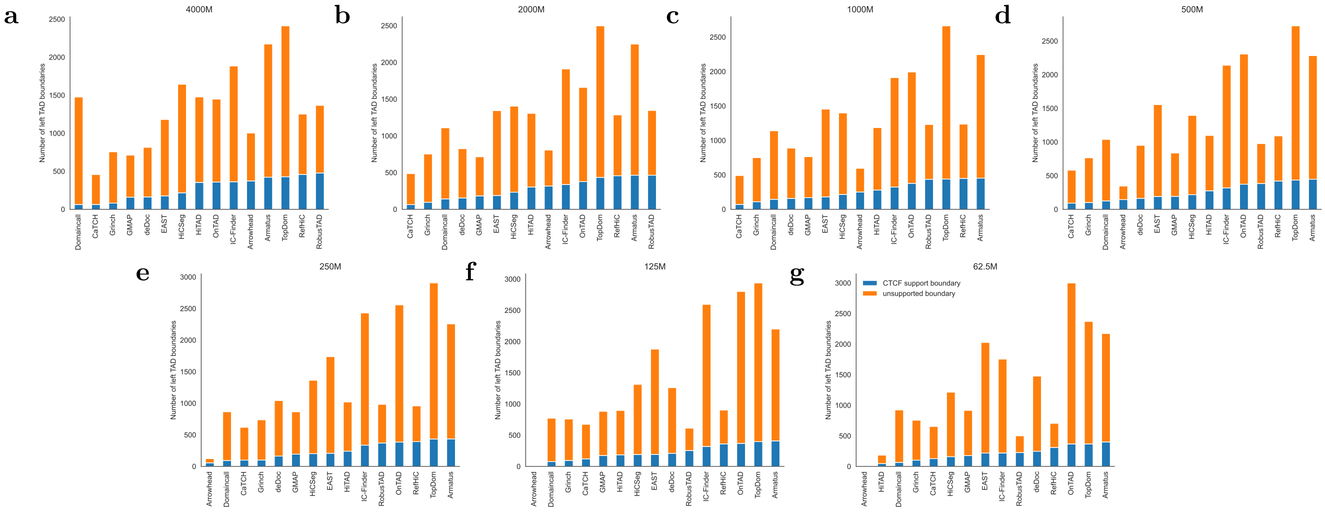

Figure S4. Number of left TAD boundaries predicted by different tools, and proportion of predicted boundaries that are supported by CTCF ChIP-Seq data.

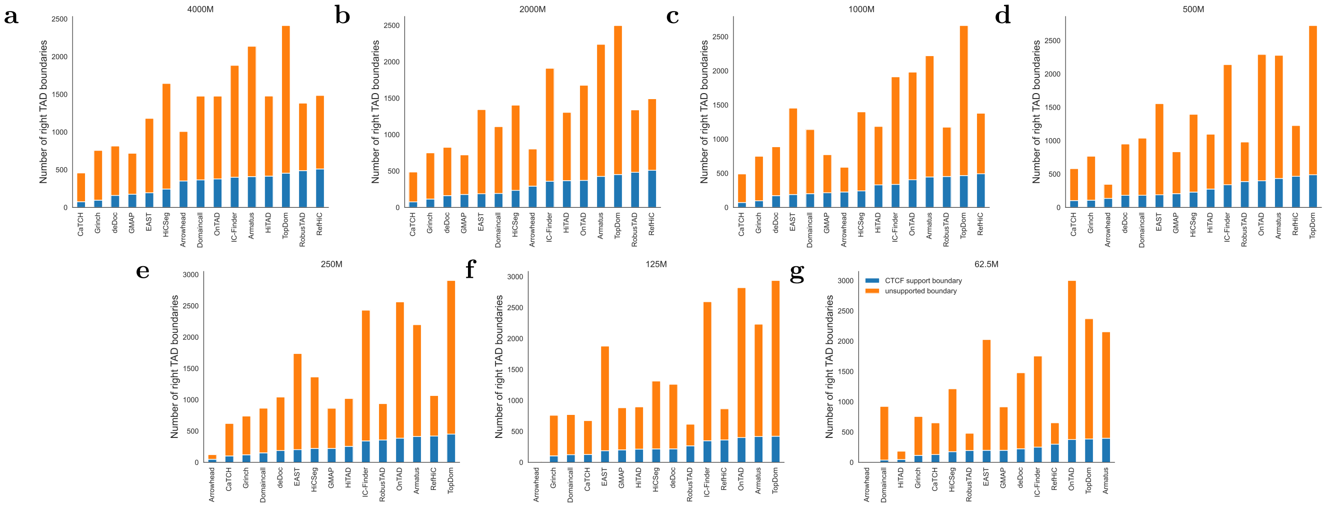

Figure S5. Number of right TAD boundaries predicted by different tools, and proportion of predicted boundaries that are supported by CTCF ChIP-Seq data.

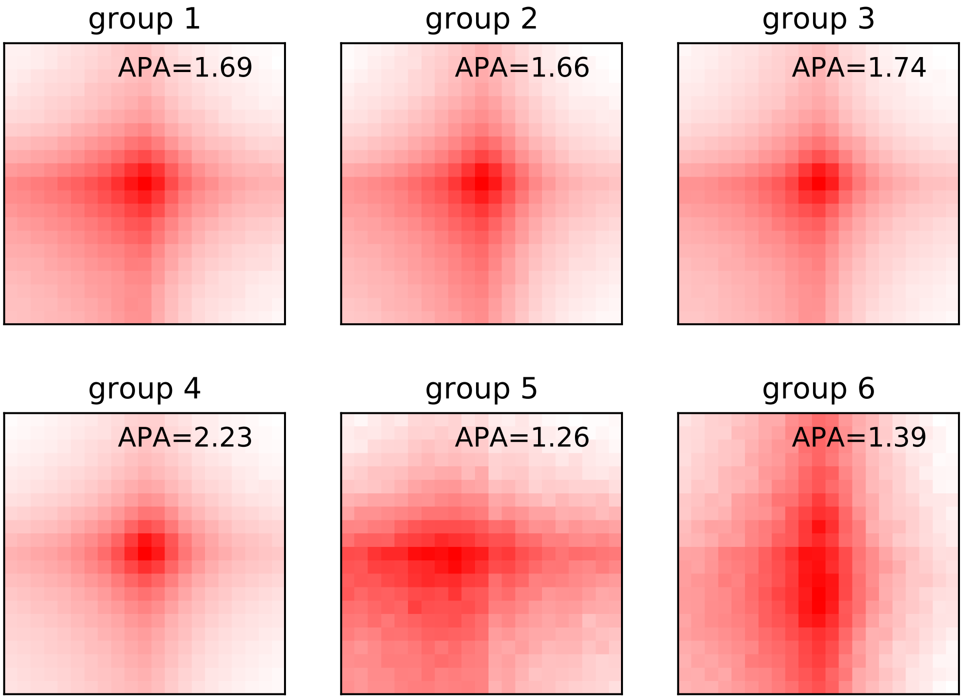

Figure S6. Aggregate peak analysis (APA) at TAD corners for each TAD group identified from the combined GM12878 Hi-C data.

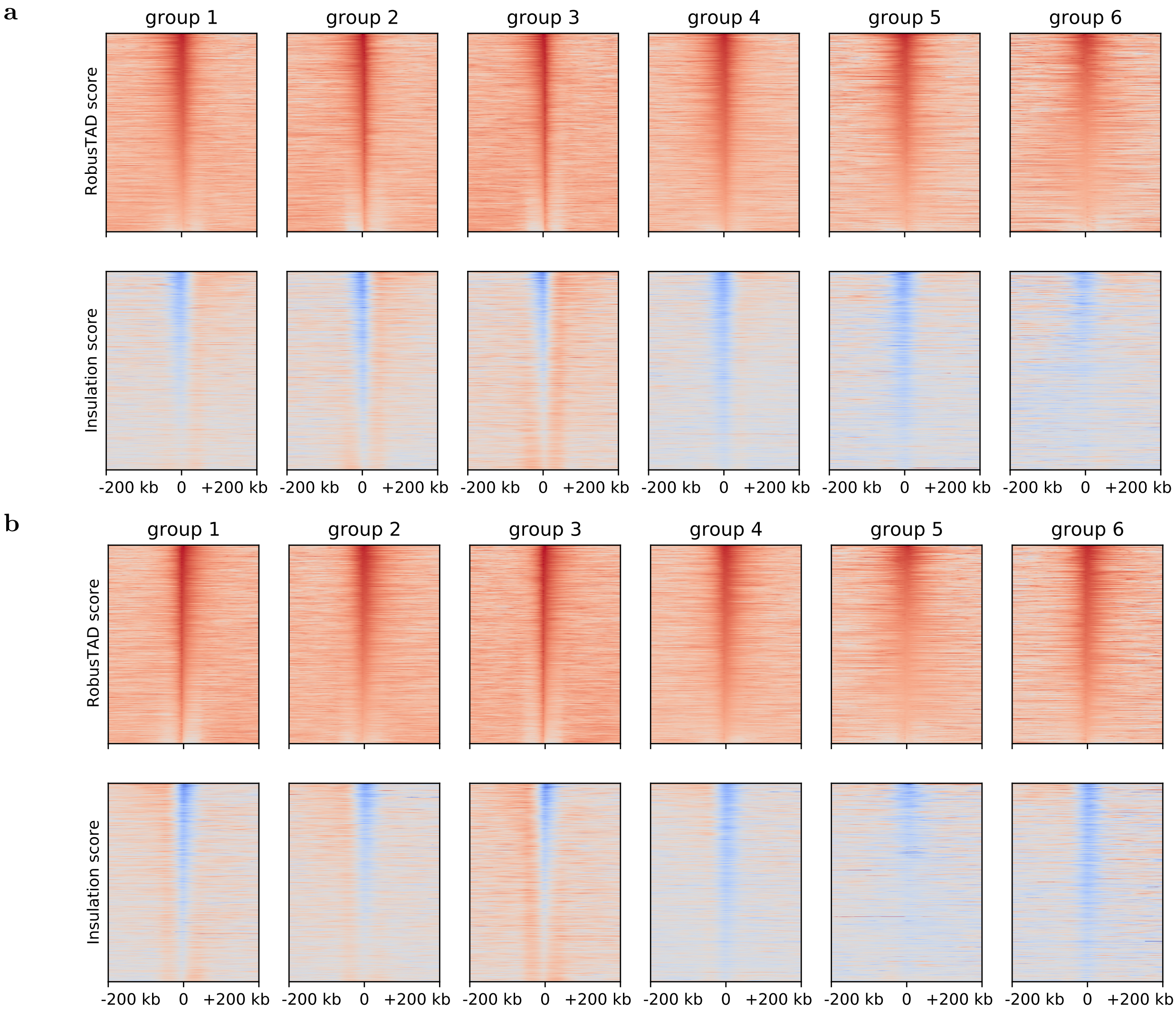

Figure S7. Insulation score and RobusTAD score around domain boundaries of the six groups of TADs predicted from the combined Hi-C data for GM12878 cells.

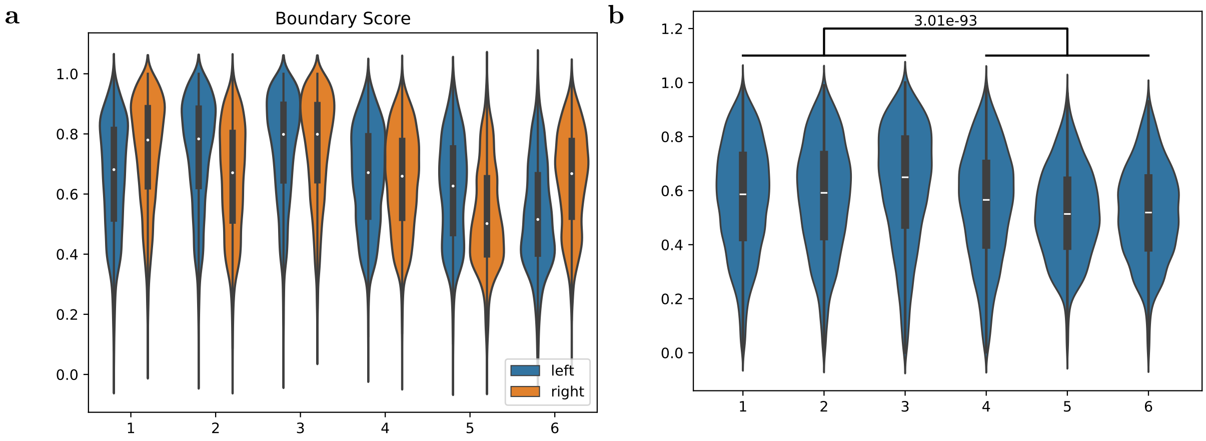

Figure S8. RobusTAD score at domain boundaries (a) and domains (b) of the six groups of TADs predicted from the combined Hi-C data for GM12878 cells. Groups 1, 2, and 3 correspond to TADs related to active regions, while groups 4, 5, and 6 correspond to TADs related to repressive regions. We quantified the differences using a two-tailed p-value test.

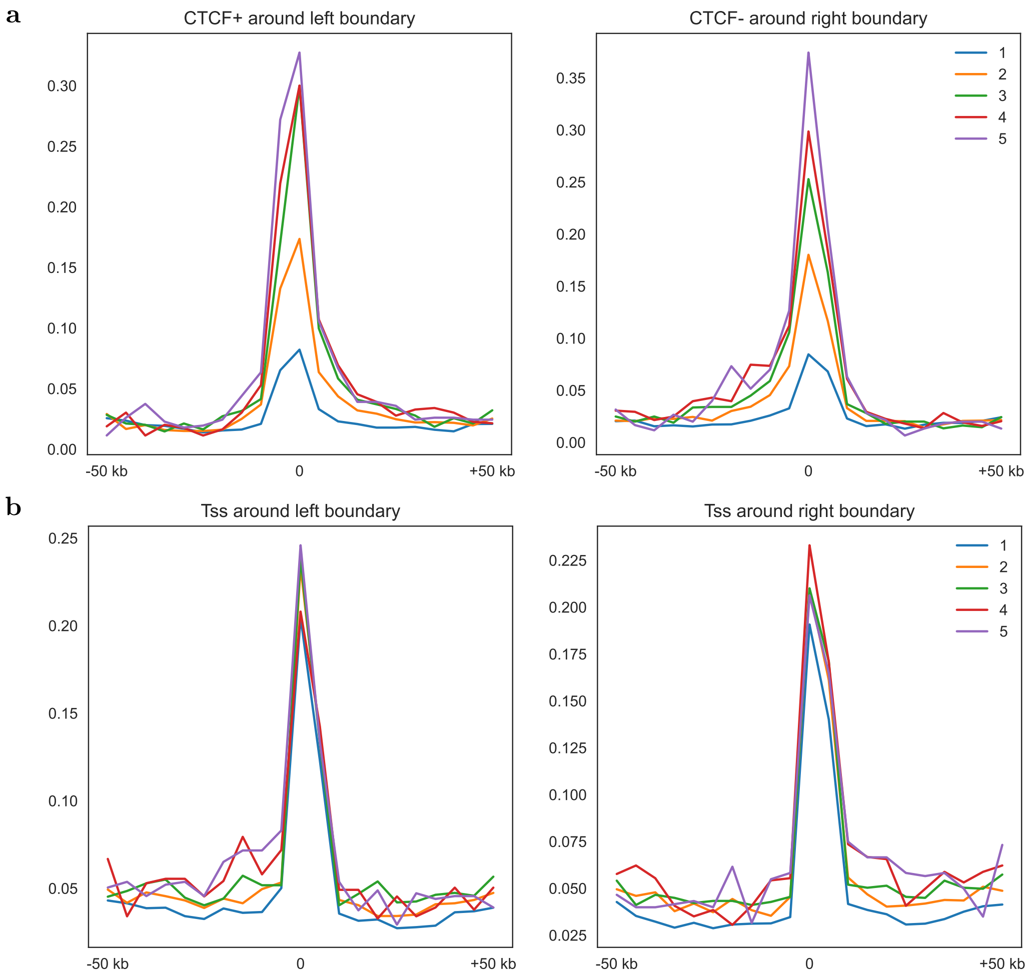

Figure S9. Enrichment of CTCF binding sites and activate promoters around domain boundaries. We classify a boundary into one of five groups based on the number of times it acts as a domain boundary for different TADs.

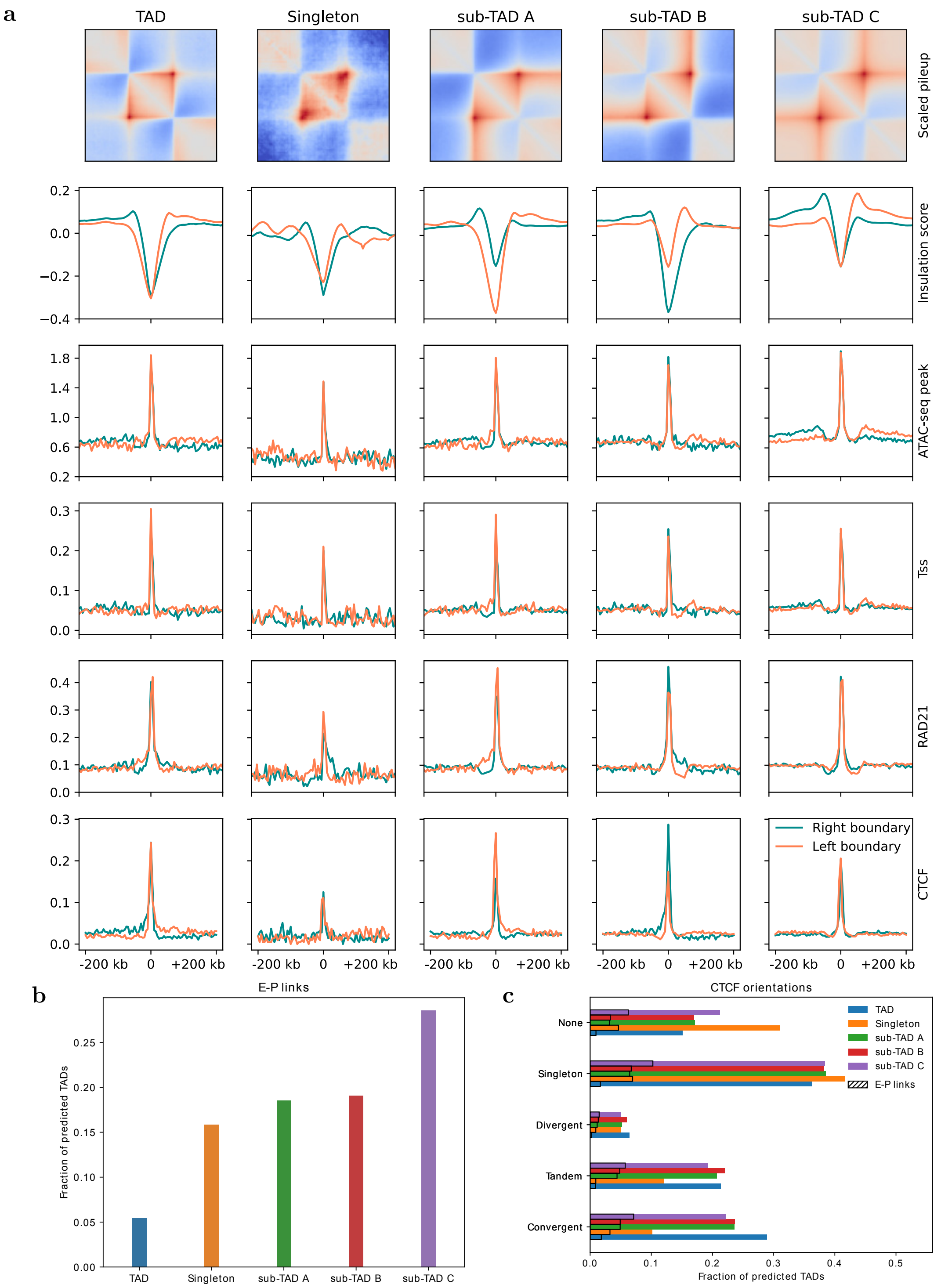

Figure S10. A comparison of singleton TADs, TADs, and subTADs. a, rescaled pileup plots around TADs, and distributions of insulation scores, ATAC-Seq peaks, Tss, RAD21, and CTCF binding sites around domain boundaries. **b,** Proportions of domains being E-P links. **c,** Orientation of CTCF motifs at TAD boundary pairs.

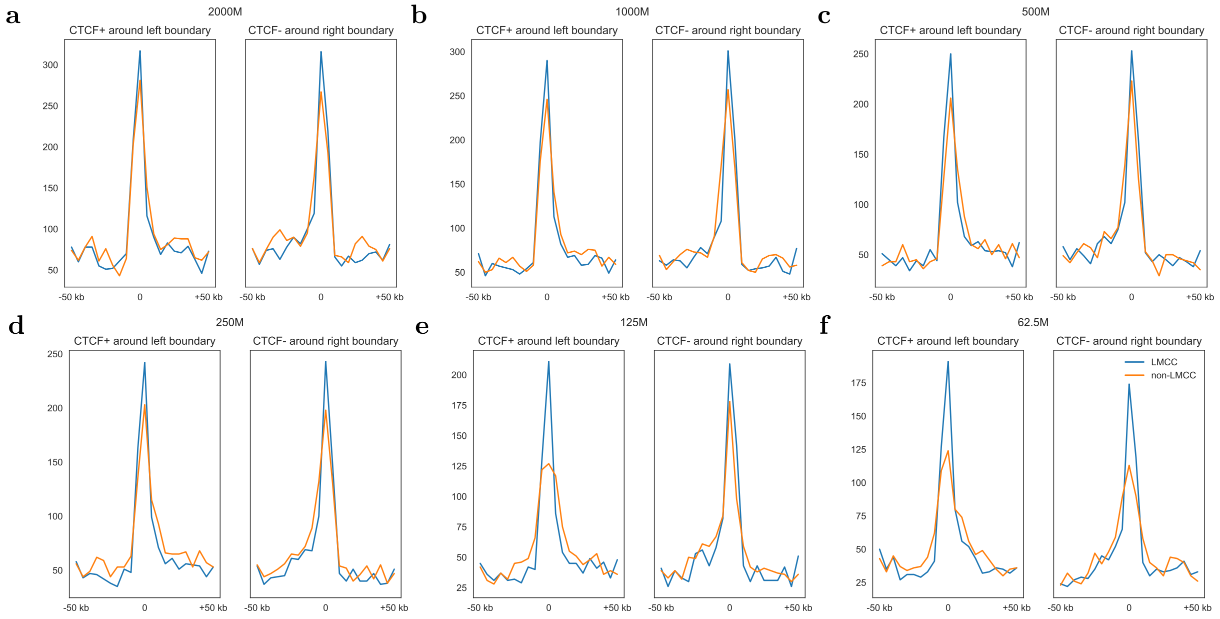

Figure S11. An accuracy comparison of domain boundaries identified by RobusTAD with and without LMCC boundary refinement. a-f show the occupancy of ChIP-seq identified CTCF binding site as a function of distance to domain boundaries that predicted from Hi-C data containing various number of contact pairs.

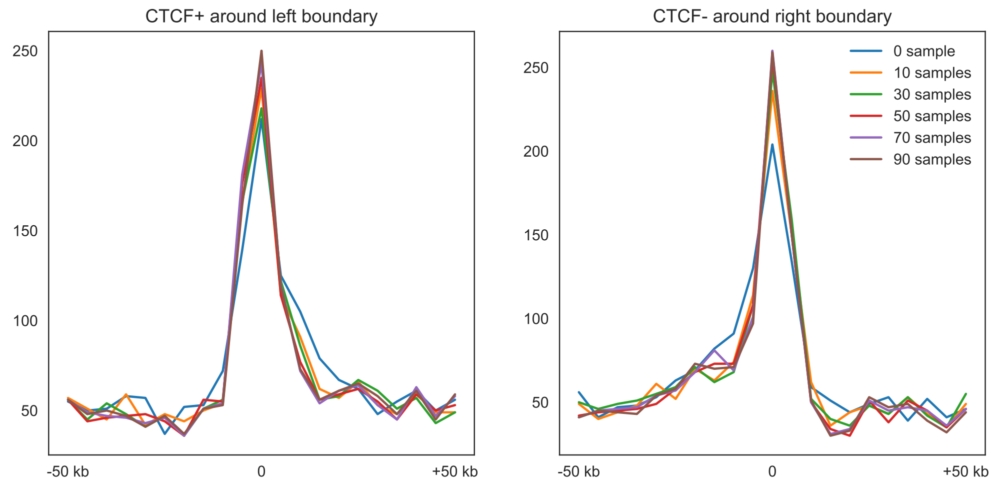

Figure S12. An accuracy comparison of domain boundaries identified by RobusTAD with different number of reference samples. The two plots show the occupancy of ChIP-seq identified CTCF binding site as a function of distance to domain boundaries that predicted from Hi-C data containing 250M valid read pairs using various samples as a reference panel.

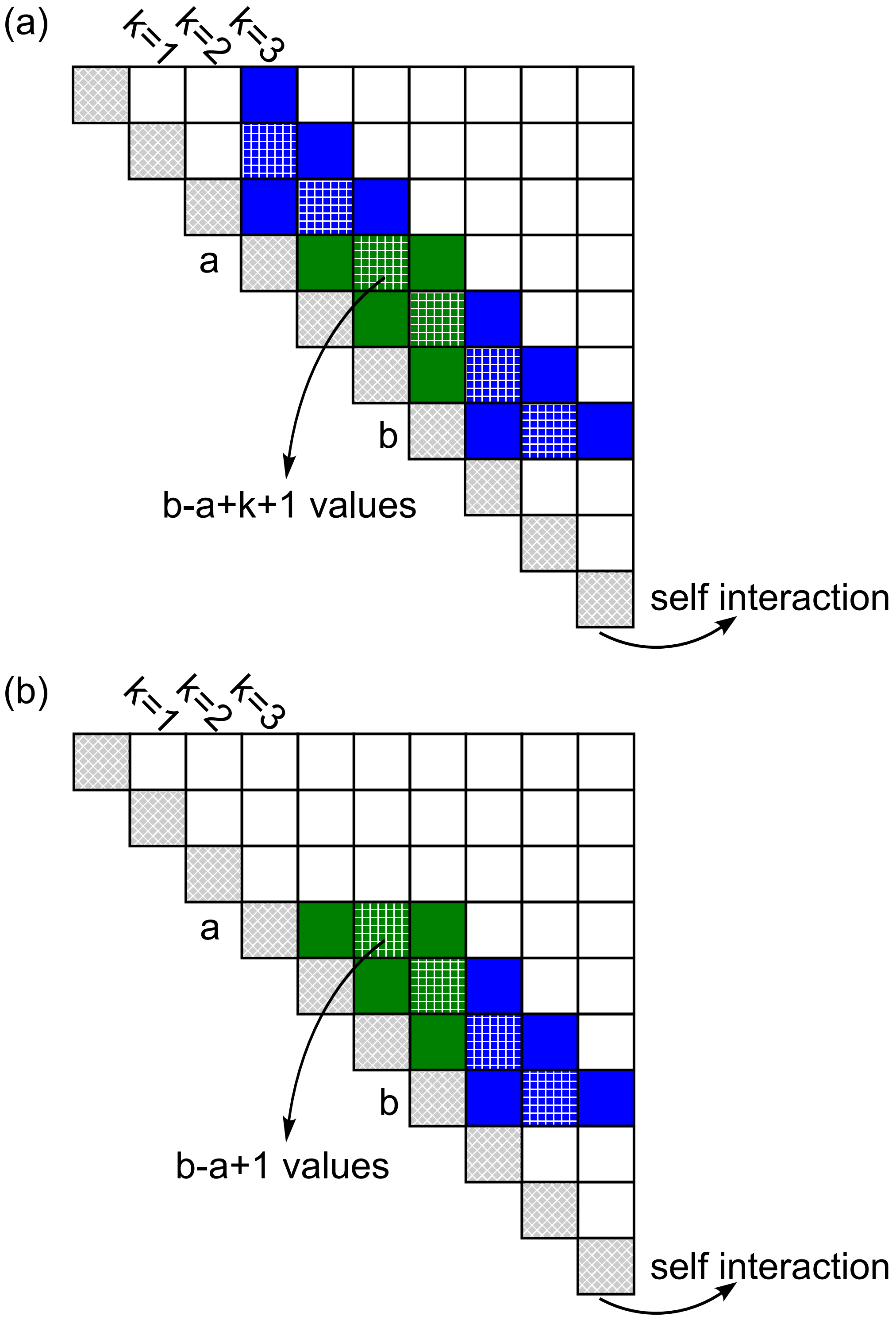

Figure S13. An example of interactions along the kth diagonal involved in the calculation of the TAD score (a) and the right boundary score (b). The green and blue regions correspond to the within-domain and across-TAD-boundary interactions, respectively. Highlighted interactions refer to the $b - a + k + 1$ and $b - a + 1$ values along the kth diagonal. Self-interactions are excluded from our calculations.

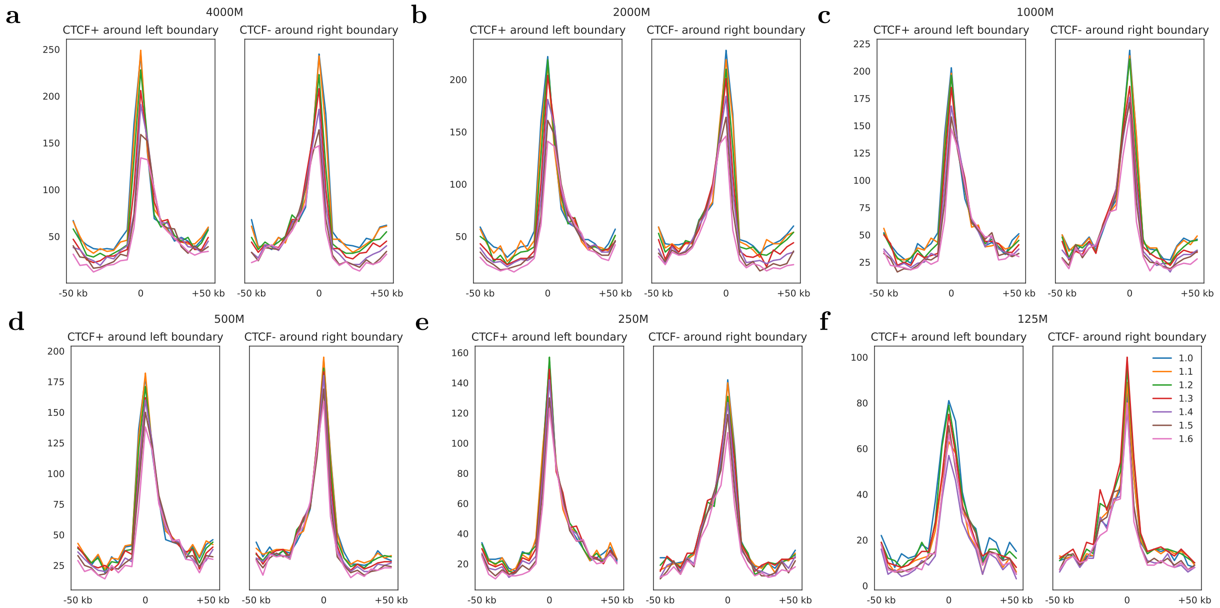

Figure S14. An accuracy comparison of domain boundaries identified by RobusTAD with different γ. a-f show the occupancy of ChIP-seq identified CTCF binding site as a function of distance to domain boundaries that predicted from Hi-C data containing various number of contact pairs.

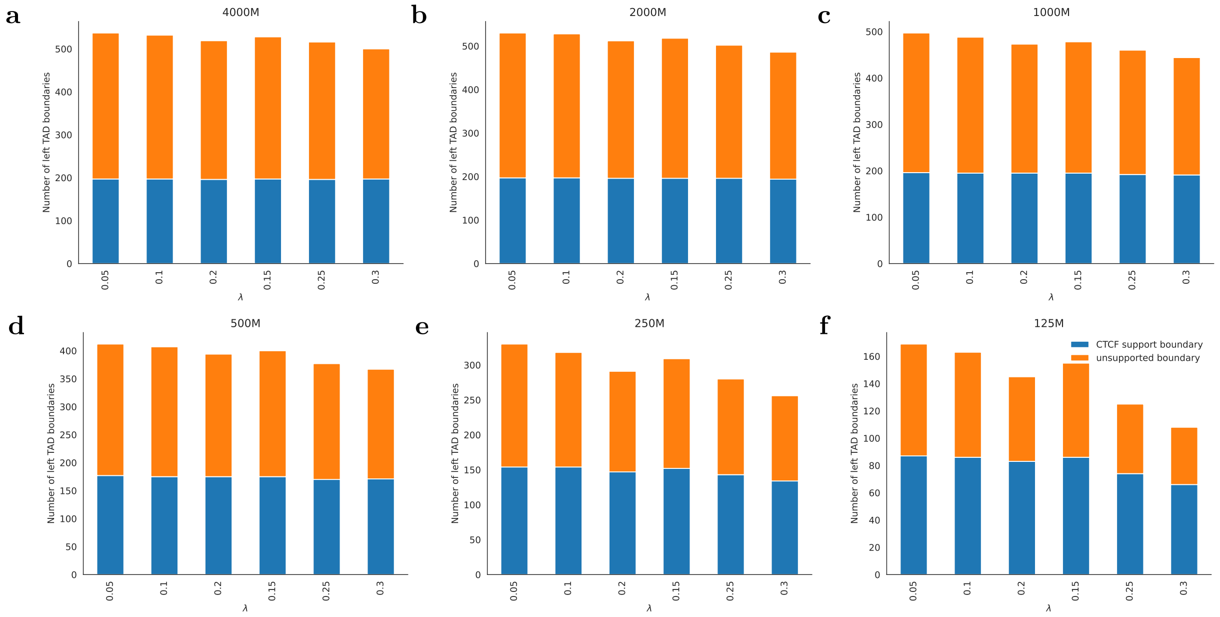

Figure S15. An accuracy comparison of TAD identified by RobusTAD with different λ. a-f show proportion of predicted TADs from Hi-C data containing various number of contact pairs that are supported by CTCF ChIA-PET data.

Table S1: Reference Panel

Accession numbers	Sample	Source
GSM3358191, GSM3358192	22Rv1 (prostate cancer cell line)	[13]
GSM3901271, GSM3901272	293TRex-Flag-BRD4-NUT-HA, treat 1 μg/mL tetracycline for 8 hours	[32]
GSM2631393, GSM2631395	786-M1A cell line (renal cancer cell line)	[31]
GSM2631392, GSM2631394	786-O cell line (renal cancer cell line)	[31]
GSM4198752, GSM4198762	BLaER (lymphoblastic leukemia cell line), CEBPA fused with the estrogen receptor (ER) hormone-binding domain, induced 120hour	[35]
GSM4198753, GSM4198763	BLaER (lymphoblastic leukemia cell line), CEBPA fused with the estrogen receptor (ER) hormone-binding domain, induced 144hour	[35]
GSM4198749, GSM4198759	BLaER (lymphoblastic leukemia cell line), CEBPA fused with the estrogen receptor (ER) hormone-binding domain, induced 48hour	[35]
GSM4198750, GSM4198760	BLaER (lymphoblastic leukemia cell line), CEBPA fused with the estrogen receptor (ER) hormone-binding domain, induced 72hour	[35]
GSM4198751, GSM4198761	BLaER (lymphoblastic leukemia cell line), CEBPA fused with the estrogen receptor (ER) hormone-binding domain, induced 96hour	[35]
GSM4198746, GSM4198756	BLaER (lymphoblastic leukemia cell line), CEBPA fused with the estrogen receptor (ER) hormone-binding domain, induced 9hour	[35]
GSM4198768, GSM4198772	BLaER (lymphoblastic leukemia cell line), CTCF-auxin inducible degradation, treat DMSO, 168hour	[35]
GSM3967131, GSM3967132	CUTLL1 (T-ALL cell lines), 1μM DMSO treat every 12h for 72h treat, Arima	[19]
GSM3967126, GSM3967127	CUTLL1 (T-ALL cell lines), 1μM DMSO treat every 12h for 72h, HindIII	[19]
GSM3967129, GSM3967130	CUTLL1 (T-ALL cell lines), 1μM γSI treat every 12 h for 72h	[19]
GSM3967124	Early T-lineage progenitor acute lymphoblastic leukemia (ETP-ALL)	[19]
GSM2825105, GSM2825106	G-401 (kidney cancer cell line)	[11]
GSM3258551	HCC1954 (Breast cancer cell line)	[2]
GSM2809575, GSM2809576, GSM2809577, GSM2809578	HCT-116 (colorectal cancer cell line), RAD21 alleles were tagged with an AID domain and a fluorescent mClover, 6hour axin treat, 180min withdrawal	[29]
GSM3898435, GSM3898437	HCT116 cell, auxin-inducible degron (AID) tag fused to STAG1, auxin treat	[8]

GSM3898434, GSM3898436	HCT116 cell, auxin-inducible degron (AID) tag fused to STAG1, no auxin treat	[8]
GSM3898439, GSM3898441	HCT116 cell, auxin-inducible degron (AID) tag fused to STAG2, auxin treat	[8]
GSM3898438, GSM3898440	HCT116 cell, auxin-inducible degron (AID) tag fused to STAG2, no auxin treat	[8]
GSM3489420	HeLa F2 cell, treated for 24 hours with 1000 U/ml of recombinant human IFNg	[30]
GSM2747750	HeLa Kyoto	[39]
GSM4106788	HeLa Kyoto cell, HindIII G1 sync control	[38]
GSM4106796	HeLa Kyoto cell, HindIII G1 sync control, CTCF and ESCO1 siRNA depleted	[38]
GSM4106802	HeLa Kyoto cell, HindIII G1 sync control, CTCF and STAG1 siRNA depleted	[38]
GSM4106795	HeLa Kyoto cell, HindIII G1 sync control, CTCF and STAG2 siRNA depleted	[38]
GSM4106794	HeLa Kyoto cell, HindIII G1 sync control, CTCF siRNA depleted	[38]
GSM4106797	HeLa Kyoto cell, HindIII G1 sync control, ESCO siRNA depleted	[38]
GSM4106792	HeLa Kyoto cell, HindIII G1 sync control, STAG1 siRNA depleted	[38]
GSM4106793	HeLa Kyoto cell, HindIII G1 sync control, STAG2 siRNA depleted	[38]
GSM4106789	HeLa Kyoto cell, MboI G1 sync control	[38]
GSM4106799	HeLa Kyoto cell, MboI G1 sync control, auxin-inducible degron (AID) tag fused to STAG1, auxin treat	[38]
GSM4106798	HeLa Kyoto cell, MboI G1 sync control, auxin-inducible degron (AID) tag fused to STAG1, no auxin treat	[38]
GSM4106801	HeLa Kyoto cell, MboI G1 sync control, auxin-inducible degron (AID) tag fused to STAG2, auxin treat	[38]
GSM4106800	HeLa Kyoto cell, MboI G1 sync control, auxin-inducible degron (AID) tag fused to STAG2, no auxin treat	[38]
GSM4106790	HeLa Kyoto cell, MboI G1 sync control, STAG1 siRNA depleted	[38]
GSM4106791	HeLa Kyoto cell, MboI G1 sync control, STAG2 siRNA depleted	[38]
GSM2747751	HeLa Kyoto, CTCF-auxin inducible degradation, 0min	[39]

GSM2747752	HeLa Kyoto, CTCF-auxin inducible degradation, 120min	[39]
GSM2747740	HeLa Kyoto, Pds5SA/B depleted by RNA inference	[39]
GSM2747745, GSM2747748	HeLa Kyoto, Scc1-auxin inducible degradation, 0min	[39]
GSM2747749	HeLa Kyoto, Scc1-auxin inducible degradation, 120min	[39]
GSM2747746	HeLa Kyoto, Scc1-auxin inducible degradation, 15min	[39]
GSM2747747	HeLa Kyoto, Scc1-auxin inducible degradation, 180min	[39]
GSM2747753	HeLa Kyoto, Scc1-auxin inducible degradation, WAPL and Pds5SA/B depleted by RNA inference, 0min	[39]
GSM2747754	HeLa Kyoto, Scc1-auxin inducible degradation, WAPL and Pds5SA/B depleted by RNA inference, 15min	[39]
GSM2747755	HeLa Kyoto, Scc1-auxin inducible degradation, WAPL and Pds5SA/B depleted by RNA inference, 180min	[39]
GSM2747738	HeLa Kyoto, synchronized at G1	[39]
GSM2747744	HeLa Kyoto, synchronized at G2	[39]
GSM2747743	HeLa Kyoto, synchronized at S	[39]
GSM2747741	HeLa Kyoto, WAPL and Pds5SA/B depleted by RNA inference	[39]
GSM2747739	HeLa Kyoto, WAPL depleted by RNA inference	[39]
GSM2825569, GSM2825570	HepG2 (hepatocellular carcinoma cell line)	[11]
GSM3304262, GSM3304264	HT1080 (fibrosarcoma cell line)	[18]
GSM2597682, GSM2597683	IMR90 (Lung fibroblast-derived myoblast), control vector	[9]
GSM2597686, GSM2597687	IMR90 (Lung fibroblast-derived myoblast), TET-inducible MYOD, differentiation media	[9]
GSM2597684, GSM2597685	IMR90 (Lung fibroblast-derived myoblast), TET-inducible MYOD, Growth media	[9]
GSM3967128	Jurkat (T-ALL cell lines), 1μM DMSO treat every 12h for 72h	[19]
GSM2599093, GSM2599094	MCF10AT1 (hyperplastic breast cell)	[12]
GSM2599095, GSM2599096	MCF10CA1a (fully malignant breast cancer cell)	[12]
GSM3336890, GSM3336891, GSM3336892	MCF-7 (endocrine-sensitive breast cancer cells), endocrine-sensitive ER+ cell	[1]
GSM3336896, GSM3336897,GSM3336898	MCF-7 (endocrine-sensitive breast cancer cells), Fulvestrant-resistant cell	[1]

GSM3756151, GSM3756152	MCF-7 (endocrine-sensitive breast cancer cells), grown without exposure to endocrine therapy, culture 3month	[1]
GSM3756153, GSM3756154	MCF-7 (endocrine-sensitive breast cancer cells), grown without exposure to endocrine therapy, culture 6month	[1]
GSM3756149, GSM3756150	MCF-7 (endocrine-sensitive breast cancer cells), grown without exposure to endocrine therapy, culture start	[1]
GSM3336893, GSM3336894, GSM3336895	MCF-7 (endocrine-sensitive breast cancer cells), Tamoxifen-resistant (TAMR) cell	[1]
GSM3211391	Nalm6 (B cell precursor leukemia cell line)	[36]
GSM3258552	OE33 (Esophegeal adenocarcinoma cell line)	[2]
GSM3967114	Peripheral blood T cells	[19]
GSM4119020, GSM4119025	Primary CD4+ T-cells	[40]
GSM4119022, GSM4119027	Primary CD4+ T-cells, CD3/CD28 stimulated, 1hr	[40]
GSM4119021, GSM4119026	Primary CD4+ T-cells, CD3/CD28 stimulated, 20min	[40]
GSM4119024	Primary CD4+ T-cells, CD3/CD28 stimulated, 24hr	[40]
GSM4119023, GSM4119028	Primary CD4+ T-cells, CD3/CD28 stimulated, 4hr	[40]
GSM3392701, GSM3392702	RMG1 (Ovarian clear cell adenocarcinoma cell line), ARID1A Knock Out	[37]
GSM3392703, GSM3392704	RMG1 (Ovarian clear cell adenocarcinoma cell line), NCAPH2 knock Down	[37]
GSM3327706	SNU16 (gastric cancer cell line)	[26]
GSM3258550	SNU-C1 (Colorectal cancer cell line)	[2]
GSM4594449	SW480 (Colorectal cancer cell line), treated with siRNA targeting TCF7L2, 72hour elapsed	[6]
GSM3399745	SW480 (Colorectal cancer cell line)	[2]
GSM3399746	SW480rep1 (Colorectal cancer cell line)	[2]
GSM3258549	SW480rep2 (Colorectal cancer cell line)	[2]
GSM3333325	T2000877 (gastric cancer cell line), CCNE1-rearranged gastric cancer cell line	[26]
GSM3044586, GSM3044588, GSM3044590	T-47D (ductal carcinoma cell line)	[3]
GSM3044586, GSM3044588, GSM3044592	T-47D (ductal carcinoma cell line), treat 110 mM NaCl, 1hour	[3]
GSM3356360	T990275 (gastric cancer cell line), CCNE1-rearranged gastric cancer cell line	[26]
GSM3735784, GSM3735785	WI38 Primary Fibrolabsts, replicative senescence - proliferative	[34]

GSM3735786, GSM3735787	WI38 Primary Fibrolabsts, replicative senescence - senescence	[34]
GSM3735782, GSM3735783	WI38 RAF (WI-38hTERT/GFP-RAF1-ER), Oncogene induces senescence day10	[34]
GSM3735776, GSM3735777	WI38 RAF (WI-38hTERT/GFP-RAF1-ER), Oncogene induces senescence day2	[34]
GSM3735778, GSM3735779	WI38 RAF (WI-38hTERT/GFP-RAF1-ER), Oncogene induces senescence day4	[34]
GSM3735788, GSM3735789	WI38 RAF (WI-38hTERT/GFP-RAF1-ER), Oncogene induces senescence day5, treat siDNMT1	[34]
GSM3735790	WI38 RAF (WI-38hTERT/GFP-RAF1-ER), Oncogene induces senescence day5, treat siNT1	[34]
GSM3735780, GSM3735781	WI38 RAF (WI-38hTERT/GFP-RAF1-ER), Oncogene induces senescence day6	[34]
GSM3735774, GSM3735775	WI38 RAF (WI-38hTERT/GFP-RAF1-ER), uninduced	[34]
GSM3417098	MCF10 cell line (ER-/PR- fibrocystic disease)	[22]
GSM3262956, GSM3262957	Embryonic stem cell, Cardiomyocyte differentiation : hESCs (day 0)	[41]
GSM3262962, GSM3262963	Embryonic stem cell, Cardiomyocyte differentiation : cardiac progenitors (day 7)	[41]
GSM3262964, GSM3262965	Embryonic stem cell, Cardiomyocyte differentiation : primitive cardiomyocytes (day 15)	[41]
GSM3262966, GSM3262967	Embryonic stem cell, Cardiomyocyte differentiation : ventricular cardiomyocytes (day 80)	[41]
GSM3263085, GSM3263086	Embryonic stem cell	[41]
GSM3263087, GSM3263088	Embryonic stem cell, HERV-H1 Knock-Out, HERV-H elements located TAD boundaries were deleted using CRISPR?Cas9	[41]
GSM3263089, GSM3263090	Embryonic stem cell, HERV-H2 Knock-Out, HERV-H elements located TAD boundaries were deleted using CRISPR?Cas9	[41]
GSM3734958, GSM3734959	Embryonic stem cell, HERV-H2-insertion clone1	[41]
GSM3734960, GSM3734961	Embryonic stem cell, HERV-H2-insertion clone2	[41]
GSM3593256, GSM3593257	teloHAEC (endothelial cell line)	[21]
GSM3593258, GSM3593259	teloHAEC (endothelial cell line), TNFα treated, 4hour	[21]
GSM3560407, GSM3560408	primary white blood cell	-
GSM3560409	primary neutrophil cell	-
GSM3438650, GSM3438651	HUVEC (umblical vein endothelial cells)	[16]
GSM3438652, GSM3438653	HUVEC (umblical vein endothelial cells), treated 10 ng/ml TNF-α, 1hour	[16]

GSM2973922, GSM2973923	ASCs (Adipose-Derived Stem Cells), 0 day of differentiation induction	[27]
GSM2973924, GSM2973925	ASCs (Adipose-Derived Stem Cells), 1 day of differentiation induction	[27]
GSM2973928, GSM2973929	ASCs (Adipose-Derived Stem Cells), 2 days before induction of differentiation	[27]
GSM2973930, GSM2973931	ASCs (Adipose-Derived Stem Cells), 1 day after neuronal induction	[27]
GSM2973932, GSM2973933	ASCs (Adipose-Derived Stem Cells), 3 day after neuronal induction	[27]
GSM2410309, GSM2410310	Naïve human embryonic stem cells, growth condition: GSKi + MEKi (2i), Lif, IGF1, FGF	[4]
GSM3506961, GSM3506962, GSM3506963, GSM3506964, GSM3506965, GSM3506966, GSM3506967, GSM3506968, GSM3506969, GSM3506970	GM23248 (primary skin fibroblasts)	[25]
GSM3112369, GSM3112370	HTBE (human tracheobronchial epithelial cells), infect active H5N1 influenza, infection time 12hour	[15]
GSM3112371, GSM3112372	HTBE (human tracheobronchial epithelial cells), infect UV-inactived H5N1 influenza, infection time 12hour	[15]
GSM3112373, GSM3112374	HTBE (human tracheobronchial epithelial cells), infect mock, infection time 12hour	[15]
GSM3112375, GSM3112376	HTBE (human tracheobronchial epithelial cells), infect active H5N1 influenza, infection time 18hour	[15]
GSM3112377, GSM3112378	HTBE (human tracheobronchial epithelial cells), infect UV-inactived H5N1 influenza, infection time 18hour	[15]
GSM3112379, GSM3112380	HTBE (human tracheobronchial epithelial cells), infect mock, infection time 18hour	[15]
GSM3112381, GSM3112382	HTBE (human tracheobronchial epithelial cells), infect active H5N1 influenza, infection time 6hour	[15]
GSM3112383, GSM3112384	HTBE (human tracheobronchial epithelial cells), infect UV-inactived H5N1 influenza, infection time 6hour	[15]
GSM3112385, GSM3112386	HTBE (human tracheobronchial epithelial cells), infect mock, infection time 6hour	[15]
GSM3112387, GSM3112388	MDM (monocyte-derived macrophages), infect active H5N1 influenza, infection time 12hour	[15]
GSM3112389, GSM3112390	MDM (monocyte-derived macrophages), infect UV-inactived H5N1 influenza, infection time 12hour	[15]

GSM3112391, GSM3112392	MDM (monocyte-derived macrophages), infect mock, infection time 12hour	[15]
GSM3112395, GSM3112396	MDM (monocyte-derived macrophages), infect UV-inactived H5N1 influenza, infection time 18hour	[15]
GSM3112397, GSM3112398	MDM (monocyte-derived macrophages), infect mock, infection time 18hour	[15]
GSM3112399, GSM3112400, GSM3111878, GSM3111879	MDM (monocyte-derived macrophages), infect active H5N1 influenza, infection time 6hour	[15]
GSM3112401, GSM3112402	MDM (monocyte-derived macrophages), infect UV-inactived H5N1 influenza, infection time 6hour	[15]
GSM3112403, GSM3112404, GSM3111876, GSM3111877	MDM (monocyte-derived macrophages), infect mock, infection time 6hour	[15]
GSM3111880, GSM3111881	MDM (monocyte-derived macrophages), infect H5N1-dNS1 influenza, infection time 6hour	[15]
GSM3111882, GSM3111883	MDM (monocyte-derived macrophages), treat IFNb, 6hour	[15]
GSM2816609, GSM2816610	H9 human Embryonic Stem Cell Line, Heat shock condition	[24]
GSM3110157, GSM3110158	MCF10a (epithelial cell line), arrested in G1	[20]
GSM3110159, GSM3110160	MCF10a (epithelial cell line), arrested in G1 and transfected STAG1 siRNA	[20]
GSM3110161, GSM3110162	MCF10a (epithelial cell line), arrested in G1 and transfected STAG2 siRNA	[20]
GSM2595581	HUVEC (Human umbilical vein endothelial cells), donor1	[42]
GSM2595583	HUVEC (Human umbilical vein endothelial cells), donor3	[42]
GSM2595584	IMR90 (fetal lung fibroblast cell), I10	[42]
GSM2595585	IMR90 (fetal lung fibroblast cell), I79	[42]
GSM2595586	MSC (mesenchymal stromal cells)	[42]
GSM2595587	HUVEC (Human umbilical vein endothelial cells), donor1, Oncogenic induced senescence	[42]
GSM2595588	HUVEC (Human umbilical vein endothelial cells), donor2, Oncogenic induced senescence	[42]
GSM2595592	MSC (mesenchymal stromal cells), Oncogenic induced senescence	[42]
GSM2845448, GSM2845449	RUES2 (Embryonic stem cells), cardiac differentiation stage : Embryonic stem cells (ESC)	[5]
GSM3452717, GSM3452718	WTC-11 (iPSCs), cardiac differentiation stage : pluripotent stem cells (PSC)	[5]
GSM2627219, GSM2627220	RWPE1 (prostate cell line)	[23]
GSM2828874, GSM2828875	endothelial of hepatic sinusoid primary cell	[11]

GSM2824366, GSM2824367	astrocyte of the cerebellum primary cell	[11]
GSM2247305, GSM2247308	primary epidermal keratinocyte, Differentiation Day 0	[33]
GSM2247306, GSM2247309	primary epidermal keratinocyte, Differentiation Day 3	[33]
GSM2247307, GSM2247310	primary epidermal keratinocyte, Differentiation Day 6	[33]
GSM2494290, GSM2494294, GSM2494298	HAP1 (near-haploid cell line)	[14]
GSM2494291, GSM2494295, GSM2494299	HAP1 (near-haploid cell line), WAPL knock Out	[14]
GSM2494292, GSM2494296, GSM2494300	HAP1 (near-haploid cell line), SSC Knock Out	[14]
GSM2494293, GSM2494297, GSM2494301	HAP1 (near-haploid cell line), WAPL and SSC Knock OUT	[14]
GSM2225739, GSM2225740	purified human naïve B cells	[7]
GSM1267198, GSM1267199	H1 Mesendoderm Cell	[10]
GSM1267200, GSM1267201	H1 Mesenchymal Stem Cell	[10]
GSM2437834, GSM2437835, GSM2437836, GSM2437837, GSM2437838, GSM2437839, GSM2437840, GSM2437841	A549 00h 100 nM dexamethasone	[11]
GSM2437749, GSM2437750, GSM2437751, GSM2437752, GSM2437753, GSM2437754, GSM2437755	A549 01h 100 nM dexamethasone	[11]
GSM2437783, GSM2437784, GSM2437785, GSM2437786, GSM2437787, GSM2437788, GSM2437789, GSM2437790	A549 04h 100 nM dexamethasone	[11]
GSM2437857, GSM2437858, GSM2437859, GSM2437860, GSM2437861, GSM2437862, GSM2437863, GSM2437864	A549 08h 100 nM dexamethasone	[11]
GSM2437806, GSM2437807, GSM2437808, GSM2437809, GSM2437810, GSM2437811, GSM2437812, GSM2437813	A549 12h 100 nM dexamethasone	[11]
GSM1551629, GSM1551630, GSM1551631	HUVEC, in-situ MboI	-

GSM1551599, GSM1551600, GSM1551601, GSM1551602, GSM1551603, GSM1551604, GSM1551605	IMR90, in-situ MboI	-
GSM1551618, GSM1551619, GSM1551620, GSM1551621, GSM1551622, GSM1551623	K562, in-situ MboI	-
GSM1551624, GSM1551625, GSM1551626, GSM1551627, GSM1551628	KBM7, in-situ MboI	[28]
GSM1551614, GSM1551615, GSM1551616	NHEK, in-situ MboI	[28]
GSM2297252, GSM2297253, GSM2297254, GSM2297255	H1-derived Mesenchymal Stem Cell	[17]

References

[1] J. Achinger-Kawecka, F. Valdes-Mora, P. L. Luu, K. A. Giles, C. E. Caldon, W. Qu, S. Nair, S. Soto, W. J. Locke, N. S. Yeo-Teh, C. M. Gould, Q. Du, G. C. Smith, I. R. Ramos, K. F. Fernandez, D. S. Hoon, J. M. W. Gee, C. Stirzaker, and S. J. Clark. Epigenetic reprogramming at estrogen-receptor binding sites alters 3D chromatin landscape in endocrine-resistant breast cancer. *Nat Commun*, 11(1):320, Jan 2020.

[2] K. C. Akdemir, V. T. Le, S. Chandran, Y. Li, R. G. Verhaak, R. Beroukhim, P. J. Campbell, L. Chin, J. R. Dixon, P. A. Futreal, et al. Disruption of chromatin folding domains by somatic genomic rearrangements in human cancer. *Nature genetics*, 52(3):294–305, 2020.

[3] R. Amat, R. ttcher, F. Le Dily, E. Vidal, J. Quilez, Y. Cuartero, M. Beato, E. de Nadal, and F. Posas. Rapid reversible changes in compartments and local chromatin organization revealed by hyperosmotic shock. *Genome Res*, 29(1):18–28, Jan 2019.

[4] S. L. Battle, N. Doni Jayavelu, R. N. Azad, J. Hesson, F. N. Ahmed, E. G. Overbey, J. A. Zoller, J. Mathieu, H. Ruohola-Baker, C. B. Ware, and R. D. Hawkins. Enhancer Chromatin and 3D Genome Architecture Changes from Naive to Primed Human Embryonic Stem Cell States. *Stem Cell Reports*, 12(5):1129–1144, May 2019.

[5] A. Bertero, P. A. Fields, V. Ramani, G. Bonora, G. G. Yardimci, H. Reinecke, L. Pabon, W. S. Noble, J. Shendure, and C. E. Murry. Dynamics of genome reorganization during human cardiogenesis reveal an RBM20-dependent splicing factory. *Nat Commun*, 10(1):1538, Apr 2019.

[6] M. A. Brown, G. A. Dotson, S. Ronquist, G. Emons, I. Rajapakse, and T. Ried. TCF7L2 silencing results in altered gene expression patterns accompanied by local genomic reorganization. *Neoplasia*, 23(2):257–269, Feb 2021.

[7] K. L. Bunting, T. D. Soong, R. Singh, Y. Jiang, W. guelin, D. W. Poloway, B. L. Swed, K. Hatzi, W. Reisacher, M. Teater, O. Elemento, and A. M. Melnick. Multi-tiered Reorganization of the Genome during B Cell Affinity Maturation Anchored by a Germinal Center-Specific Locus Control Region. *Immunity*, 45(3):497–512, Sep 2016.

[8] V. Casa, M. Moronta Gines, E. Gade Gusmao, J. A. Slotman, A. Zirkel, N. Josipovic, E. Oole, W. F. J. van IJcken, A. B. Houtsmuller, A. Papantonis, and K. S. Wendt. Redundant and specific roles of cohesin STAG subunits in chromatin looping and transcriptional control. *Genome Res*, 30(4):515–527, Apr 2020.

[9] A. Dall'Agnese, L. Caputo, C. Nicoletti, J. di Iulio, A. Schmitt, S. Gatto, Y. Diao, Z. Ye, M. Forcato, R. Perera, S. Bicciato, A. Telenti, B. Ren, and P. L. Puri. Transcription Factor-Directed Re-wiring of Chromatin Architecture for Somatic Cell Nuclear Reprogramming toward trans-Differentiation. *Mol Cell*, 76(3):453–472, Nov 2019.

[10] J. R. Dixon, I. Jung, S. Selvaraj, Y. Shen, J. E. Antosiewicz-Bourget, A. Y. Lee, Z. Ye, A. Kim, N. Rajagopal, W. Xie, Y. Diao, J. Liang, H. Zhao, V. V. Lobanenkov, J. R. Ecker, J. A. Thomson, and B. Ren. Chromatin architecture reorganization during stem cell differentiation. *Nature*, 518(7539):331–336, Feb 2015.

[11] I. Dunham, A. Kundaje, S. F. Aldred, P. J. Collins, C. A. Davis, F. Doyle, C. B. Epstein, S. Frietze, J. Harrow, R. Kaul, J. Khatun, and B. R. e. a. Lajoie. An integrated encyclopedia of DNA elements in the human genome. *Nature*, 489(7414):57–74, Sep 2012.

[12] A. J. Fritz, P. N. Ghule, J. R. Boyd, C. E. Tye, N. A. Page, D. Hong, D. J. Shirley, A. S. Weinheimer, A. R. Barutcu, D. L. Gerrard, S. Frietze, A. J. van Wijnen, S. K. Zaidi, A. N. Imbalzano, J. B. Lian, J. L. Stein, and G. S. Stein. Intranuclear and higher-order chromatin organization of the major histone gene cluster in breast cancer. *J Cell Physiol*, 233(2):1278–1290, Feb 2018.

[13] Y. Guo, A. A. Perez, D. J. Hazelett, G. A. Coetzee, S. K. Rhie, and P. J. Farnham. CRISPR-mediated deletion of prostate cancer risk-associated CTCF loop anchors identifies repressive chromatin loops. *Genome Biol*, 19(1):160, Oct 2018.

[14] J. H. I. Haarhuis, R. H. van der Weide, V. A. Blomen, J. O. ez Cuna, M. Amendola, M. S. van Ruiten, P. H. L. Krijger, H. Teunissen, R. H. Medema, B. van Steensel, T. R. Brummelkamp, E. de Wit, and B. D. Rowland. The Cohesin Release Factor WAPL Restricts Chromatin Loop Extension. *Cell*, 169(4):693–707, May 2017.

[15] S. Heinz, L. Texari, M. G. B. Hayes, M. Urbanowski, M. W. Chang, N. Givarkes, A. Rialdi, K. M. White, R. A. Albrecht, L. Pache, I. Marazzi, A. a Sastre, M. L. Shaw, and C. Benner. Transcription Elongation Can Affect Genome 3D Structure. *Cell*, 174(6):1522–1536, Sep 2018.

[16] Y. Higashijima, Y. Matsui, T. Shimamura, R. Nakaki, N. Nagai, S. Tsutsumi, Y. Abe, V. M. Link, M. Osaka, M. Yoshida, R. Watanabe, T. Tanaka, A. Taguchi, M. Miura, X. Ruan, G. Li, T. Inoue, M. Nangaku, H. Kimura, T. Furukawa, H. Aburatani,

Y. Wada, Y. Ruan, C. K. Glass, and Y. Kanki. Coordinated demethylation of H3K9 and H3K27 is required for rapid inflammatory responses of endothelial cells. *EMBO J*, 39(7):e103949, Apr 2020.

[17] I. Jung, A. Schmitt, Y. Diao, A. J. Lee, T. Liu, D. Yang, C. Tan, J. Eom, M. Chan, S. Chee, Z. Chiang, C. Kim, E. Masliah, C. L. Barr, B. Li, S. Kuan, D. Kim, and B. Ren. A compendium of promoter-centered long-range chromatin interactions in the human genome. *Nat Genet*, 51(10):1442–1449, Oct 2019.

[18] O. L. Kantidze, A. V. Luzhin, E. V. Nizovtseva, A. Safina, M. E. Valieva, A. K. Golov, A. K. Velichko, A. V. Lyubitelev, A. V. Feofanov, K. V. Gurova, V. M. Studitsky, and S. V. Razin. The anti-cancer drugs curaxins target spatial genome organization. *Nat Commun*, 10(1):1441, Mar 2019.

[19] A. Kloetgen, P. Thandapani, P. Ntziachristos, Y. Ghebrechristos, S. Nomikou, C. Lazaris, X. Chen, H. Hu, S. Bakogianni, J. Wang, Y. Fu, F. Boccalatte, H. Zhong, E. Paietta, T. Trimarchi, Y. Zhu, P. Van Vlierberghe, G. G. Inghirami, T. Lionnet, I. Aifantis, and A. Tsirigos. Three-dimensional chromatin landscapes in T cell acute lymphoblastic leukemia. *Nat Genet*, 52(4):388–400, Apr 2020.

[20] A. Kojic, A. Cuadrado, M. De Koninck, D. nez Llorente, M. guez Corsino, G. pez, F. Le Dily, M. A. Marti-Renom, and A. Losada. Distinct roles of cohesin-SA1 and cohesin-SA2 in 3D chromosome organization. *Nat Struct Mol Biol*, 25(6):496–504, Jun 2018.

[21] S. Lalonde, V. A. Codina-Fauteux, S. M. de Bellefon, F. Leblanc, M. Beaudoin, M. M. Simon, R. Dali, T. Kwan, K. S. Lo, T. Pastinen, and G. Lettre. Integrative analysis of vascular endothelial cell genomic features identifies AIDA as a coronary artery disease candidate gene. *Genome Biol*, 20(1):133, Jul 2019.

[22] F. Le Dily, E. Vidal, Y. Cuartero, J. Quilez, A. S. Nacht, G. P. Vicent, J. Carbonell-Caballero, P. Sharma, J. L. as, R. Ferrari, L. I. De Llobet, G. Verde, R. H. G. Wright, and M. Beato. Hormone-control regions mediate steroid receptor-dependent genome organization. *Genome Res*, 29(1):29–39, Jan 2019.

[23] Z. Luo, S. K. Rhie, F. D. Lay, and P. J. Farnham. A Prostate Cancer Risk Element Functions as a Repressive Loop that Regulates HOXA13. *Cell Rep*, 21(6):1411–1417, Nov 2017.

[24] X. Lyu, M. J. Rowley, and V. G. Corces. Architectural Proteins and Pluripotency Factors Cooperate to Orchestrate the Transcriptional Response of hESCs to Temperature Stress. *Mol Cell*, 71(6):940–955, Sep 2018.

[25] G. Nir, I. Farabella, C. rez Estrada, C. G. Ebeling, B. J. Beliveau, H. M. Sasaki, S. D. Lee, S. C. Nguyen, R. B. McCole, S. Chattoraj, J. Erceg, J. AlHaj Abed, N. M. C. Martins, H. Q. Nguyen, M. A. Hannan, S. Russell, N. C. Durand, S. S. P. Rao, J. Y. Kishi, P. Soler-Vila, M. Di Pierro, J. N. Onuchic, S. P. Callahan, J. M. Schreiner, J. A. Stuckey, P. Yin, E. L. Aiden, M. A. Marti-Renom, and C. T. Wu. Walking along

chromosomes with super-resolution imaging, contact maps, and integrative modeling. *PLoS Genet*, 14(12):e1007872, Dec 2018.

[26] W. F. Ooi, A. M. Nargund, K. J. Lim, S. Zhang, M. Xing, A. Mandoli, J. Q. Lim, S. W. T. Ho, Y. Guo, X. Yao, S. J. Lin, T. Nandi, C. Xu, X. Ong, M. Lee, A. L. Tan, Y. N. Lam, J. X. Teo, A. Kaneda, K. P. White, W. K. Lim, S. G. Rozen, B. T. Teh, S. Li, A. J. Skanderup, and P. Tan. enhancer hijacking in primary gastric adenocarcinoma. *Gut*, 69(6):1039–1052, Jun 2020.

[27] J. Paulsen, T. M. Liyakat Ali, M. Nekrasov, E. Delbarre, M. O. Baudement, S. Kurscheid, D. Tremethick, and P. Collas. Long-range interactions between topologically associating domains shape the four-dimensional genome during differentiation. *Nat Genet*, 51(5):835–843, May 2019.

[28] S. S. Rao, M. H. Huntley, N. C. Durand, E. K. Stamenova, I. D. Bochkov, J. T. Robinson, A. L. Sanborn, I. Machol, A. D. Omer, E. S. Lander, and E. L. Aiden. A 3D map of the human genome at kilobase resolution reveals principles of chromatin looping. *Cell*, 159(7):1665–1680, Dec 2014.

[29] S. S. P. Rao, S. C. Huang, B. Glenn St Hilaire, J. M. Engreitz, E. M. Perez, K. R. Kieffer-Kwon, A. L. Sanborn, S. E. Johnstone, G. D. Bascom, I. D. Bochkov, X. Huang, M. S. Shamim, J. Shin, D. Turner, Z. Ye, A. D. Omer, J. T. Robinson, T. Schlick, B. E. Bernstein, R. Casellas, E. S. Lander, and E. L. Aiden. Cohesin Loss Eliminates All Loop Domains. *Cell*, 171(2):305–320, Oct 2017.

[30] R. Raviram, P. P. Rocha, V. M. Luo, E. Swanzey, E. R. Miraldi, E. B. Chuong, C. Feschotte, R. Bonneau, and J. A. Skok. Analysis of 3D genomic interactions identifies candidate host genes that transposable elements potentially regulate. *Genome Biol*, 19(1):216, Dec 2018.

[31] P. Rodrigues, S. A. Patel, L. Harewood, I. Olan, E. Vojtasova, S. E. Syafruddin, M. N. Zaini, E. K. Richardson, J. Burge, A. Y. Warren, G. D. Stewart, K. Saeb-Parsy, S. A. Samarajiwa, and S. Vanharanta. B-Dependent Lymphoid Enhancer Co-option Promotes Renal Carcinoma Metastasis. *Cancer Discov*, 8(7):850–865, Jul 2018.

[32] C. D. Rosencrance, H. N. Ammouri, Q. Yu, T. Ge, E. J. Rendleman, S. A. Marshall, and K. P. Eagen. Chromatin Hyperacetylation Impacts Chromosome Folding by Forming a Nuclear Subcompartment. *Mol Cell*, 78(1):112–126, Apr 2020.

[33] A. J. Rubin, B. C. Barajas, M. Furlan-Magaril, V. Lopez-Pajares, M. R. Mumbach, I. Howard, D. S. Kim, L. D. Boxer, J. Cairns, M. Spivakov, S. W. Wingett, M. Shi, Z. Zhao, W. J. Greenleaf, A. Kundaje, M. Snyder, H. Y. Chang, P. Fraser, and P. A. Khavari. Lineage-specific dynamic and pre-established enhancer-promoter contacts cooperate in terminal differentiation. *Nat Genet*, 49(10):1522–1528, Oct 2017.

[34] S. Sati, B. Bonev, Q. Szabo, D. Jost, P. Bensadoun, F. Serra, V. Loubiere, G. L. Papadopoulos, J. C. Rivera-Mulia, L. Fritsch, P. Bouret, D. Castillo, J. L. Gelpi, M. Orozco, C. Vaillant, F. Pellestor, F. Bantignies, M. A. Marti-Renom, D. M. Gilbert,

J. M. Lemaitre, and G. Cavalli. 4D Genome Rewiring during Oncogene-Induced and Replicative Senescence. *Mol Cell*, 78(3):522–538, May 2020.

[35] G. Stik, E. Vidal, M. Barrero, S. Cuartero, M. s, J. Mendieta-Esteban, T. V. Tian, J. Choi, C. Berenguer, A. Abad, B. Borsari, F. le Dily, P. Cramer, M. A. Marti-Renom, R. Stadhouders, and T. Graf. CTCF is dispensable for immune cell transdifferentiation but facilitates an acute inflammatory response. *Nat Genet*, 52(7):655–661, Jul 2020.

[36] L. Tian, Y. Shao, S. Nance, J. Dang, B. Xu, X. Ma, Y. Li, B. Ju, L. Dong, S. Newman, X. Zhou, P. Schreiner, E. Tseng, T. Hon, M. Ashby, C. Li, J. Easton, T. A. Gruber, and J. Zhang. Long-read sequencing unveils IGH-DUX4 translocation into the silenced IGH allele in B-cell acute lymphoblastic leukemia. *Nat Commun*, 10(1):2789, Jun 2019.

[37] S. Wu, N. Fatkhutdinov, L. Rosin, J. M. Luppino, O. Iwasaki, H. Tanizawa, H. Y. Tang, A. V. Kossenkov, A. Gardini, K. I. Noma, D. W. Speicher, E. F. Joyce, and R. Zhang. ARID1A spatially partitions interphase chromosomes. *Sci Adv*, 5(5):eaaw5294, May 2019.

[38] G. Wutz, R. Ladurner, B. G. St Hilaire, R. R. Stocsits, K. Nagasaka, B. Pignard, A. Sanborn, W. Tang, C. rnai, M. P. Ivanov, S. Schoenfelder, P. van der Lelij, X. Huang, G. rnberger, E. Roitinger, K. Mechtler, I. F. Davidson, P. Fraser, E. Lieberman-Aiden, and J. M. Peters. from WAPL. *Elife*, 9, Feb 2020.

[39] G. Wutz, C. rnai, K. Nagasaka, D. A. Cisneros, R. R. Stocsits, W. Tang, S. Schoenfelder, G. Jessberger, M. Muhar, M. J. Hossain, N. Walther, B. Koch, M. Kueblbeck, J. Ellenberg, J. Zuber, P. Fraser, and J. M. Peters. Topologically associating domains and chromatin loops depend on cohesin and are regulated by CTCF, WAPL, and PDS5 proteins. *EMBO J*, 36(24):3573–3599, Dec 2017.

[40] J. Yang, A. McGovern, P. Martin, K. Duffus, X. Ge, P. Zarrineh, A. P. Morris, A. Adamson, P. Fraser, M. Rattray, and S. Eyre. Analysis of chromatin organization and gene expression in T cells identifies functional genes for rheumatoid arthritis. *Nat Commun*, 11(1):4402, Sep 2020.

[41] Y. Zhang, T. Li, S. Preissl, M. L. Amaral, J. D. Grinstein, E. N. Farah, E. Destici, Y. Qiu, R. Hu, A. Y. Lee, S. Chee, K. Ma, Z. Ye, Q. Zhu, H. Huang, R. Fang, L. Yu, J. C. Izpisua Belmonte, J. Wu, S. M. Evans, N. C. Chi, and B. Ren. Transcriptionally active HERV-H retrotransposons demarcate topologically associating domains in human pluripotent stem cells. *Nat Genet*, 51(9):1380–1388, Sep 2019.

[42] A. Zirkel, M. Nikolic, K. Sofiadis, J. P. Mallm, C. A. Brackley, H. Gothe, O. Drechsel, C. Becker, J. ller, N. Josipovic, T. Georgomanolis, L. Brant, J. Franzen, M. Koker, E. G. Gusmao, I. G. Costa, R. T. Ullrich, W. Wagner, V. Roukos, P. rnberg, D. Marenduzzo, K. Rippe, and A. Papantonis. HMGB2 Loss upon Senescence Entry Disrupts Genomic Organization and Induces CTCF Clustering across Cell Types. *Mol Cell*, 70(4):730–744, May 2018.
